# Maximizing avertable doses with a minimum amount of waste for remediation of land areas around typical single family houses after radioactive fallout based on Monte Carlo simulations

**DOI:** 10.1038/s41598-021-84103-1

**Published:** 2021-02-25

**Authors:** Yvonne Hinrichsen, Robert Finck, Johan Martinsson, Christopher Rääf

**Affiliations:** Department of Translational Medicine, Medical Radiation Physics, Lund University, Malmö, 205 02 Sweden

**Keywords:** Mathematics and computing, Nuclear physics

## Abstract

The uncontrolled release of long-lived radioactive substances from nuclear accidents can contaminate inhabited land areas. The removal of topsoil is an important method for reducing future radiation exposure but can also generate a large amount of waste that needs safe disposal. To the best of our knowledge, previous studies have determined the optimal depth of topsoil removal but not the size of the area designated for this measure. For this purpose, this study performed Monte Carlo simulations of hypothetical ^137^Cs surface contamination on various ground areas in a typical northern European suburban area. The goal was to study the size of the areas needed and amount of waste generated to achieve a certain relative and absolute dose reduction. The results showed that removing the topsoil from areas larger than 3000 m^2^ around the houses in the study neighbourhood results in only marginal reduction in radiation exposure. If, on average, 5 cm of topsoil is removed over 3000 m^2^, then 150 m^3^ of waste would be generated. However, in this scenario adjacent properties benefit from each other’s decontamination, leading to a smaller amount of waste for a given reduction in future radiation exposure per inhabitant of these dwellings. Additionally, it was shown that topsoil removal over limited areas has a higher impact on the absolute dose reduction at an observation point inside or outside the houses with higher initial dose.

## Introduction

The uncontrolled release of long-lived radioactive substances from nuclear accidents can contaminate land areas and require extensive remediation measures. This was the case for the Chernobyl accident in Ukraine in 1986 and the Fukushima accident in Japan in 2011, where land contamination of ^137^Cs (half-life 30.05 years) posed a major problem^1^. Rainfall during the dispersion process increases radioactive contamination compared to what occurs in dry weather^2^. All outdoor surfaces become contaminated, although roofs, streets, trees and shrubs are cleaned by the precipitation to some extent, which leads to an accumulation of contamination on the ground. To reduce future radiation exposure, it may be necessary to remove contaminated material. This is especially important around dwellings in inhabited areas.

The ‘Inhabited Areas Handbook’^3^ was developed to assist decision and policy makers in the implementation of remediation for future situations with radioactive fallout. For dwellings, the decontamination of roofs and the surrounding vegetation and ground areas are important measures to reduce future radiation exposure. In particular, decontamination of the ground by removing the top layer of soil can be used to remove large amounts of the contaminants and thus reduce the public’s radiation exposure to a large extent; however, this action generates a large amount of waste that needs safe disposal. We chose to study this problem due to the importance of topsoil removal in reducing future radiation exposure. Roed et al.^4^ determined that removing 5 cm of topsoil is optimal for reducing the dose rate with moderate adverse impact on the area. This is also the recommendation given in the ‘Inhabited Areas Handbook’.

As far as we know, no studies have examned the optimal size for this decontamination measure based on reducing future radiation exposure and minimizing the amount of generated waste. For this reason, our previous study developed the isodose concept^5^, which determines the dose contributions for various locations from progressively increasing contaminated surfaces. The method uses the contaminated ground around a dwelling as source for external exposure, such as inside a house for example. This concept was applied to typical northern European single-family houses^6^ and was furthermore extended to a typical northern European suburban area^7^ in follow-up studies. The aim of this study is to find optimum sizes of areas for topsoil removal around northern European single-family houses with respect to the reduction of future radiation exposure and waste generation; thus, we also seek to minimize monetary costs and the adverse effects on the environment.

## Results

### Waste generation per relative reduction of future external radiation exposure

**Figure 1:**
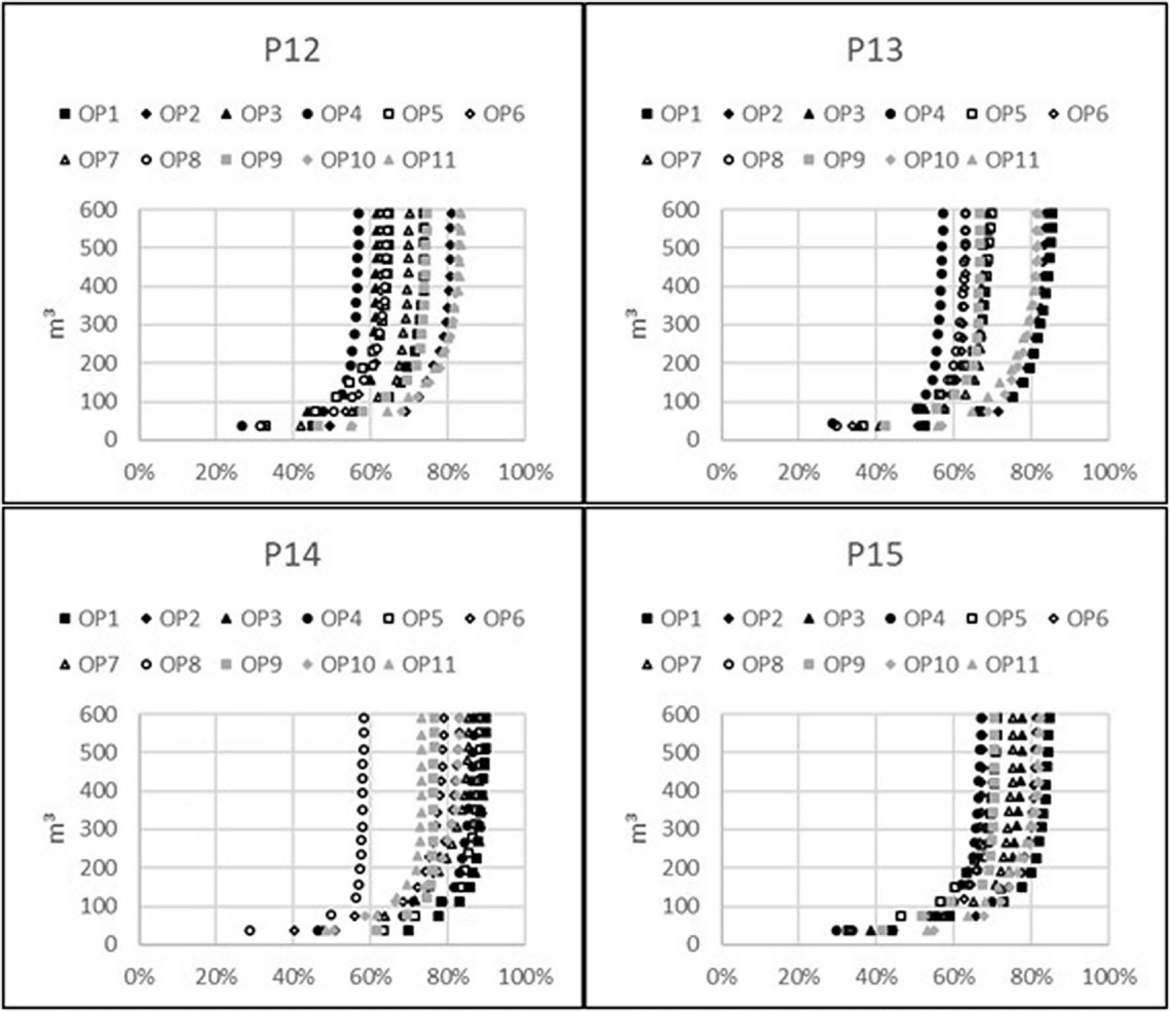
Volume of generated waste caused by topsoil removal with regard to relative dose reduction for 11 observation points inside a brick house on properties P12–P15.

**Figure 2:**
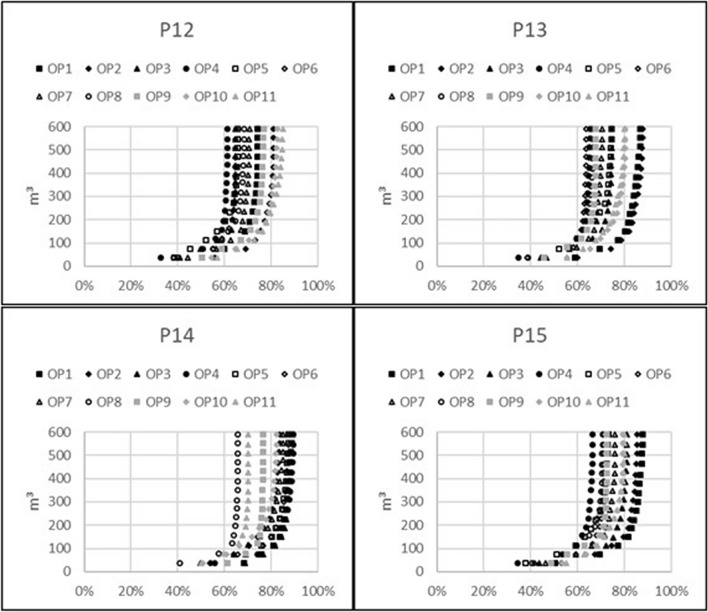
Volume of generated waste caused by topsoil removal with regard to relative dose reduction for 11 observation points inside a wooden house on properties P12–P15.

**Figure 3:**
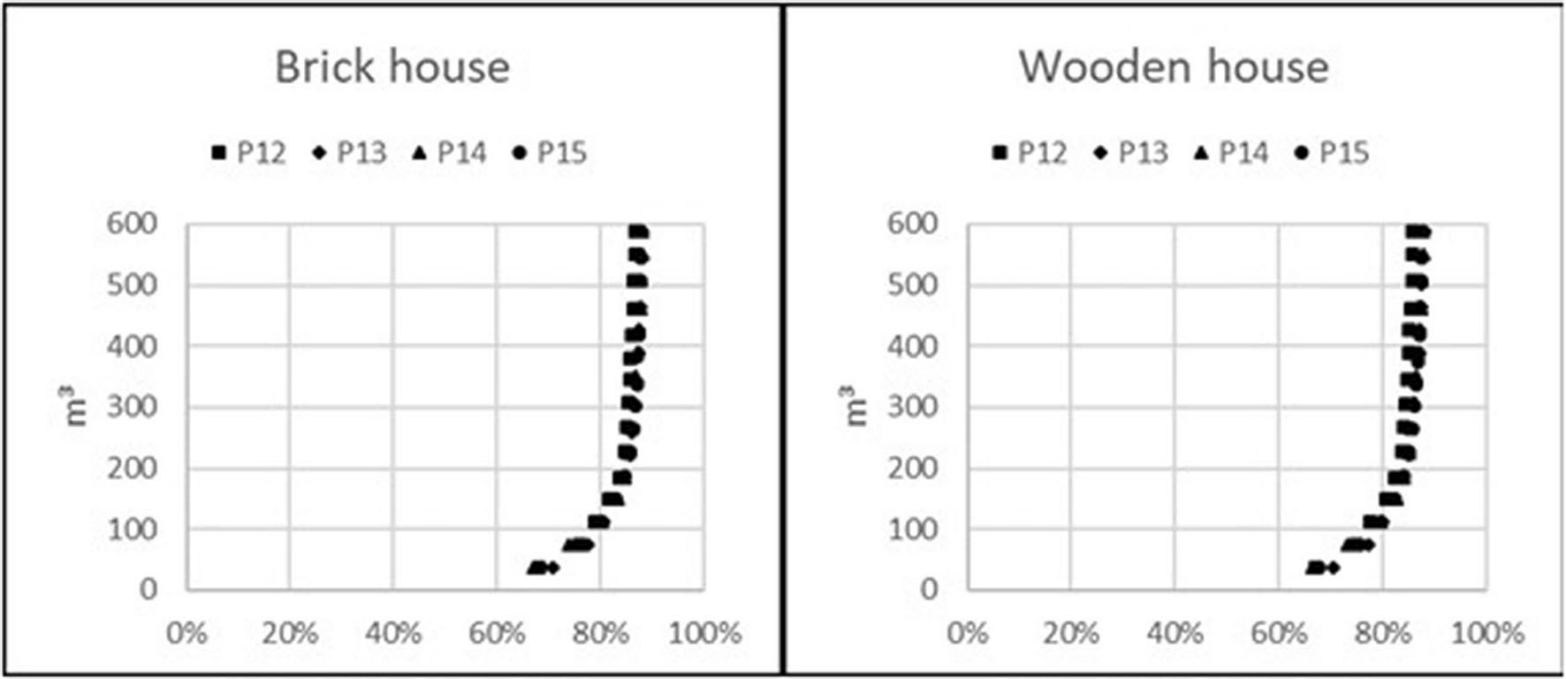
Volume of generated waste caused by topsoil removal with regard to relative dose reduction for observation points outside a brick or a wooden house on properties P12–P15.

Graphs describing the amount of waste generated by topsoil removal in relation to the relative dose reduction are presented for the 11 indoor observation points. Figure 1 is for the model including only brick houses, Fig. 2 is for the model including only wooden houses, and Fig. 3 is for the outdoor observation points in both models. In general, the resulting graphs show that waste generation of more than 150 m^3^, which equals the removal of 5 cm of topsoil over an area of 3000 m^2^, results in very little additional dose reduction, whereas the achievable relative dose reduction with reasonable amounts of generated waste is highly dependent on the observation point and varies between 55 and 90%. By studying the position of the observation points with respect to the possible achievable relative dose reduction, it can be seen that lower values are connected with open surrounding areas within the line of sight, whereas higher values occur where there are other houses within the line of sight to the open surrounding areas that shield the radiation emitted from those areas.

Furthermore, the graphs show that topsoil removal on a property covering an area of 850 m^2^ on average and generating 42.5 m^3^ of waste on average leads to a reduction between 25 and 65% of future external radiation exposure from contaminants on the ground for a situation where the fallout is on top of the soil. The exact figure depends on the observation point in the house and whether it is a wooden or brick house.

It is virtually impossible to reduce future radiation exposure caused by contamination on the ground by more than 80% on average. A higher reduction would require removing the topsoil from extensively larger areas, generating much more waste, yet this would still not achieve more than 90% dose reduction at the most favourable observation point. This is caused by the free average path length in air of about 100 m for gamma photons that are being emitted by a metastable isomer of ^137^Ba as a product of 94.4% of the beta minus decays of ^137^Cs. If the fallout area is large, there will always be dose contributions from remote areas. However, in a real fallout situation, dose contributions from other contaminated surfaces also need to be taken into consideration.

Moreover, it should be mentioned that the results presented here are based on a model where ^137^Cs is superficially distributed on the ground. Caused by surface roughness and migration of the contaminant into the ground, the soil will more effectively shield radiation from remote areas than from nearby areas^8^. This means that smaller areas around buildings need to be decontaminated to achieve a reasonable dose reduction, which also has been theoretically shown in one of our previous studies^6^.

### Decrease in radiation exposure with respect to waste generation

**Figure 4:**
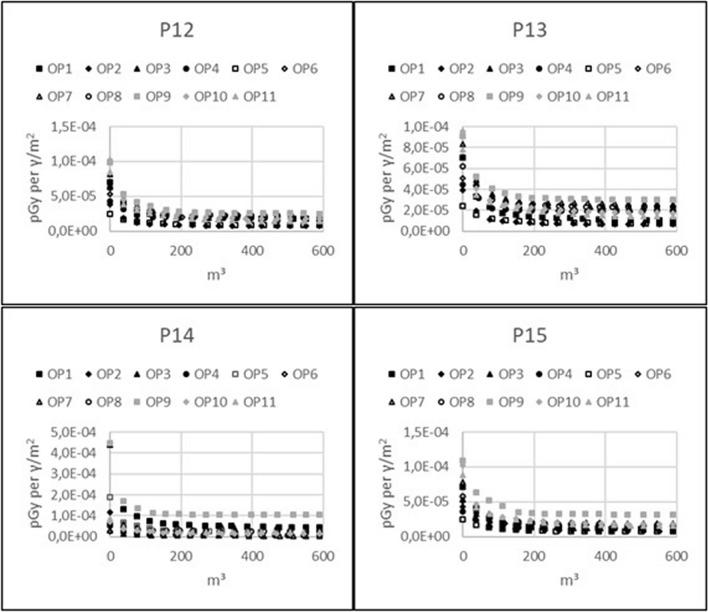
Reduction of absolute radiation exposure per volume of generated waste caused by topsoil removal for 11 observation points inside a brick house on properties P12–P15.

**Figure 5:**
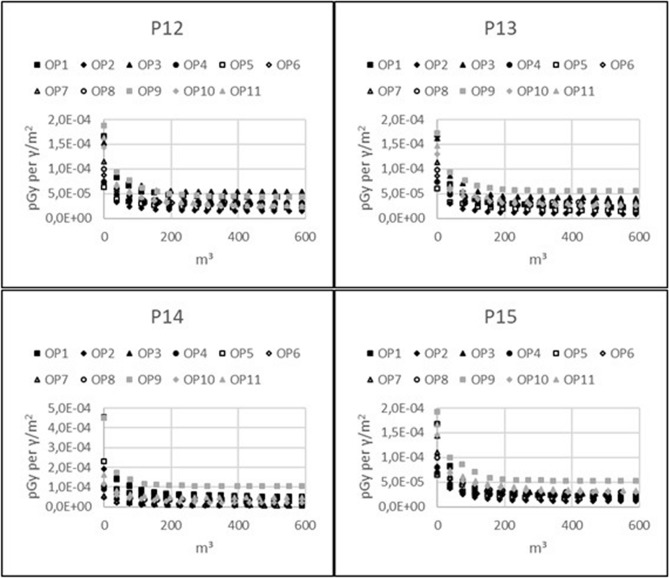
Reduction of absolute radiation exposure per volume of generated waste caused by topsoil removal for 11 observation points inside a wooden house on properties P12–P15.

**Figure 6:**
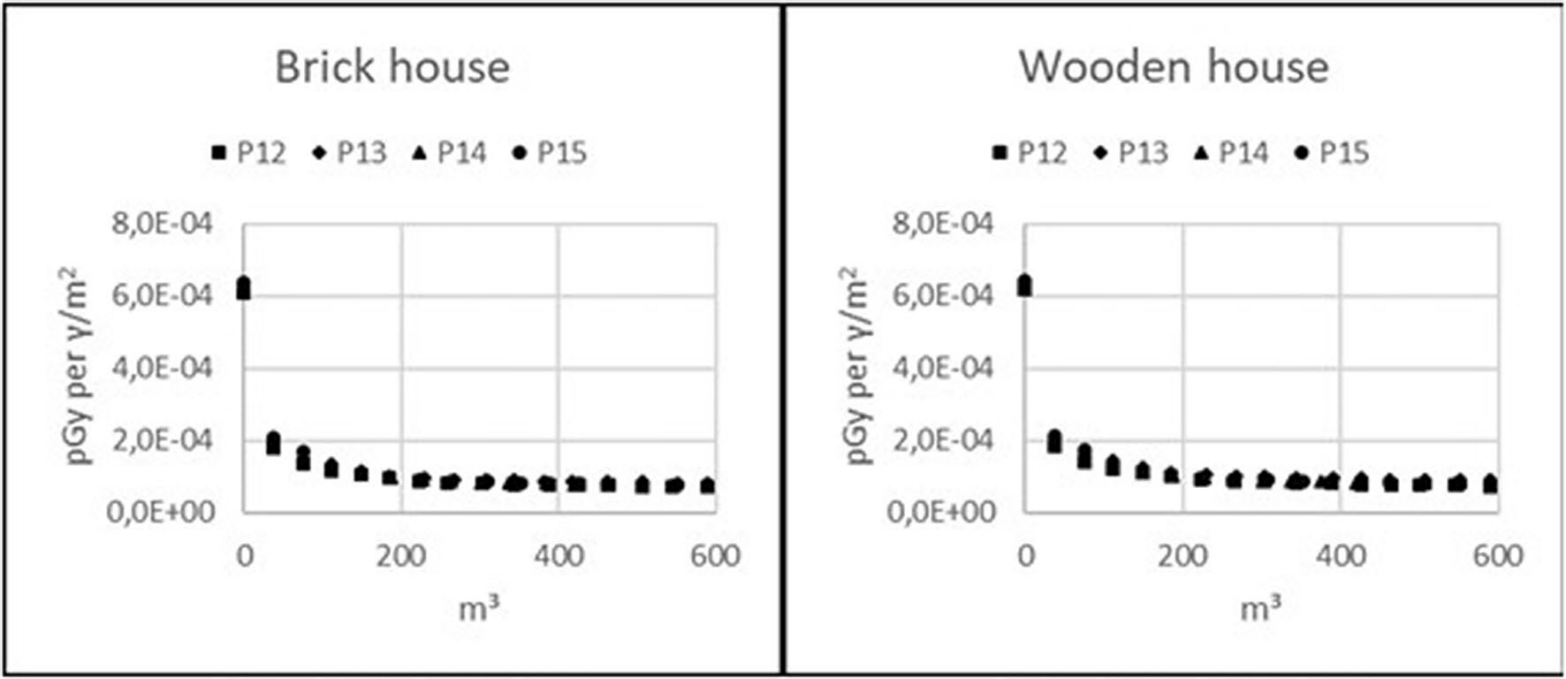
Reduction of absolute radiation exposure per volume of generated waste caused by topsoil removal for observation points outside a brick or a wooden house on properties P12–P15.

The following figures show the decrease in future radiation exposure from ground contamination with respect to waste generated by topsoil removal: Figure 4, 11 observation points inside 4 brick houses; Fig. 5, 11 observation points inside 4 wooden houses; and Fig. 6, outdoor observation points for both brick and wooden houses. The graphs show the dose contribution from the remaining non-decontaminated ground areas, expressed in units of pGy per $\gamma$/m^2^, with respect to the volume of waste. These plots can be used to illustrate how incremental reductions in dose can be achieved at observation points when decontaminating increasingly larger areas around the buildings. The gain in dose reduction gradually declines with the decontamination of more properties. The plots also show that observation points with higher initial dose will have a higher absolute dose reduction for the same amount of removed soil.

## Discussion

The results show that the external radiation exposure from ground contamination can be reduced around 60–80% by decontamination of 3000 m^2^ of ground area around houses in a typical northern European neighbourhood with property sizes of 875–1050 m^2^ and a house covering 150 m^2^. This area of 3000 m^2^ corresponds to 150 m^3^ of generated waste if 5 cm of the topsoil is removed on average. Decontamination of larger areas does not result in any significant further reduction in radiation exposure; however, it increases the amount of waste considerably and thus does not appear to be justified unless the decontamination results in a relevant reduction of radiation exposure at other observation points. The maximum eligible area that should be decontaminated is larger than the area of an individual property in this neighbourhood scenario; thus, adjacent properties will benefit from each other’s decontamination. This results in a reduced amount of waste with respect to a reduction in future radiation exposure per inhabitant of these dwellings. Therefore, decontaminating contiguous areas within a group of buildings can be advantageous.

Second, it was shown that topsoil removal over a limited area has a higher impact on the absolute dose reduction at observation points inside or outside houses with higher initial dose compared to other observation points. This is in agreement with the one of the outcomes of our previous studies^5^. In the latter study, an inverse correlation was found between the initial dose and the size of the area that must be decontaminated to achieve a certain dose reduction.

Furthermore, in connection with one of our previous studies^6^, it needs to be mentioned that, in this case, the upper extreme case was modelled in terms of dose contributions from remote areas. In reality, surface roughness and the migration of contaminants need to be taken into consideration; thus, radioactive contaminants from more remote areas will contribute less to the radiation exposure since the covering soil provides shielding. This can result in smaller areas around one dwelling that need to be decontaminated to achieve the same reduction in radiation exposure in comparison with a superficial contamination as modelled in this study. However, in practice, one also needs to consider whether the inhabitants spend their time indoors or outdoors.

Moreover, in a real fallout situation, it would be difficult to achieve reductions in radiation exposure caused by contamination of the ground that are more than around 80% on average for indoor observation points, as the dose contribution from radioactive contaminants at more remote areas is less feasible. In addition, the dose contribution from other contaminated surfaces needs to be included. However, this knowledge can support the decision maker in prioritizing areas for decontamination measures, although as shown in our previous studies^6,7^, the occupancy of the residents must also be considered.

## Methods

**Figure 7:**
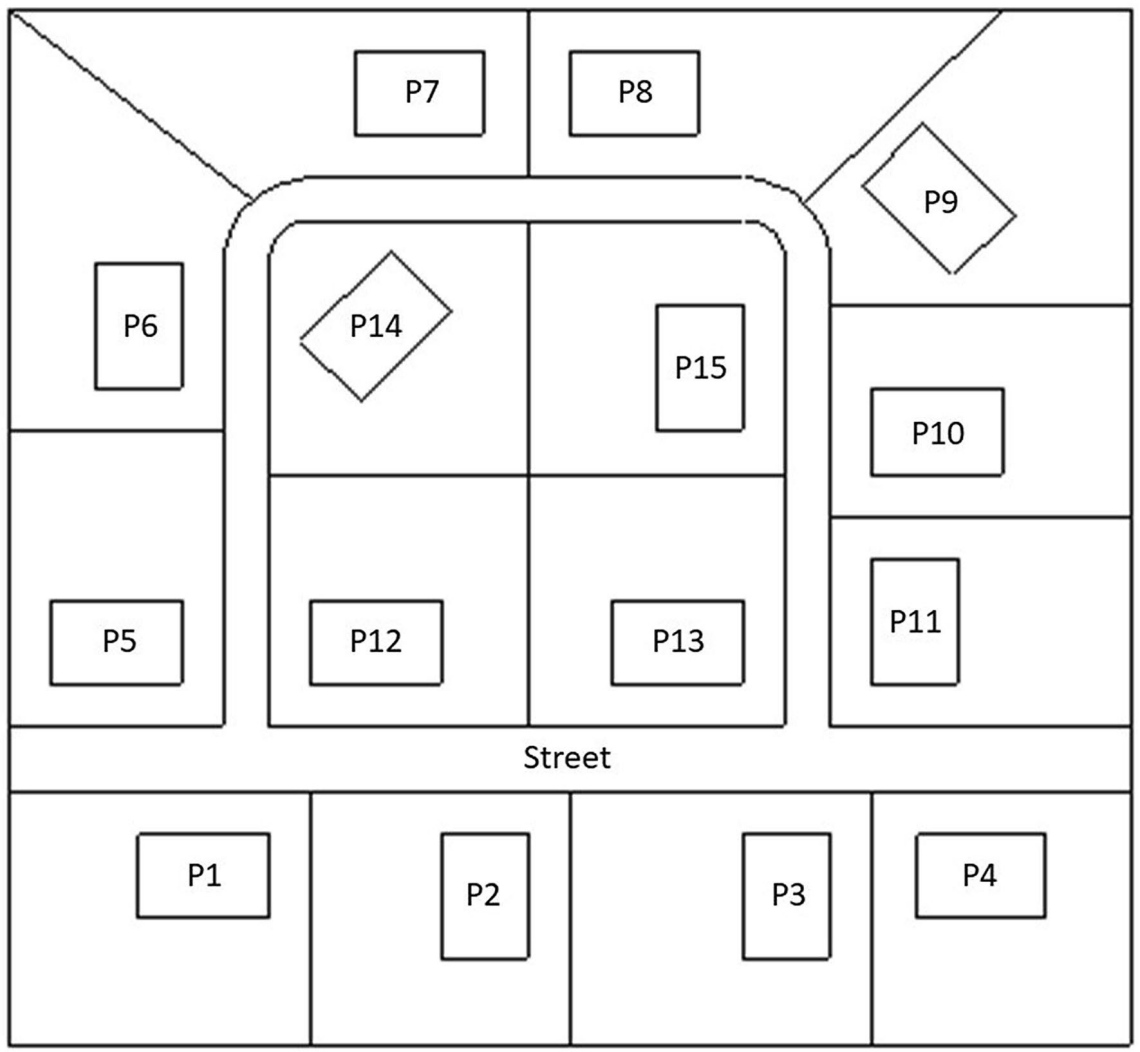
Overview of a typical Swedish suburban neighbourhood consisting of 15 properties (P1–P15), showing their respective garden boundaries and the street^7^.

Monte Carlo simulations were performed using the transport code MCNP 6.2^9^ and the nuclear cross section data set ENDF/B-VII.0^10^. Models of an actual wooden and brick house were set up in a previous study^6^ based on construction drawings made available by the Urban Planning Department of the Municipality of Hässleholm in Sweden (Stadsbyggnadskontoret, Hässleholms kommun). These models were extended to mimic a typical northern European suburban area of 16,250 m^2^. The area encompassed 15 properties (one model including only brick houses and one model including only wooden houses) of 875–1050 m^2^ each and a connecting street of ca. 1173 m^2^ (Fig. 7) in a follow-up study^7^. The results in this study are based on the same model. As each of the houses covers an area of 150 m^2^, in total, ca. 12,827 m^2^ are assumed to be unpaved (79%). Further details about the model of the houses and entire neighbourhood, including material compositions, can be found in our previous studies^6,7^.

The gamma-emitting source was defined as in our previous study^7^. An energy of 0.662 MeV represents the gamma rays being emitted by a metastable isomer of ^137^Ba as a product of 94.4% of the beta minus decays of the fission product ^137^Cs. It was chosen, because it is the most important radionuclide with respect to the long-term effects of radioactive contamination in the environment after the Chernobyl and Fukushima nuclear power plant accidents (e.g. by Imanaka et al.^1^). Furthermore, it is also among the important radionuclides with large existing sources that could be dispersed in a terrorist attack^11^. We defined the source regions at ground surface level, since the focus of this study was the effect of topsoil removal as a decontamination measure to reduce radiation exposure. Ground penetration was not considered, since one of our previous studies^6^ found that a source at this level represents an upper extreme in terms of dose contributions from areas remote to the observation. Furthermore, the optimal depth for topsoil removal has already been studied (e.g. by Roed et al.^4^). The shapes of the source areas were defined according to the gardens (excluding the area covered by the houses) and the street (Fig. 7), and an infinite area surrounded the defined area.

**Figure 8:**
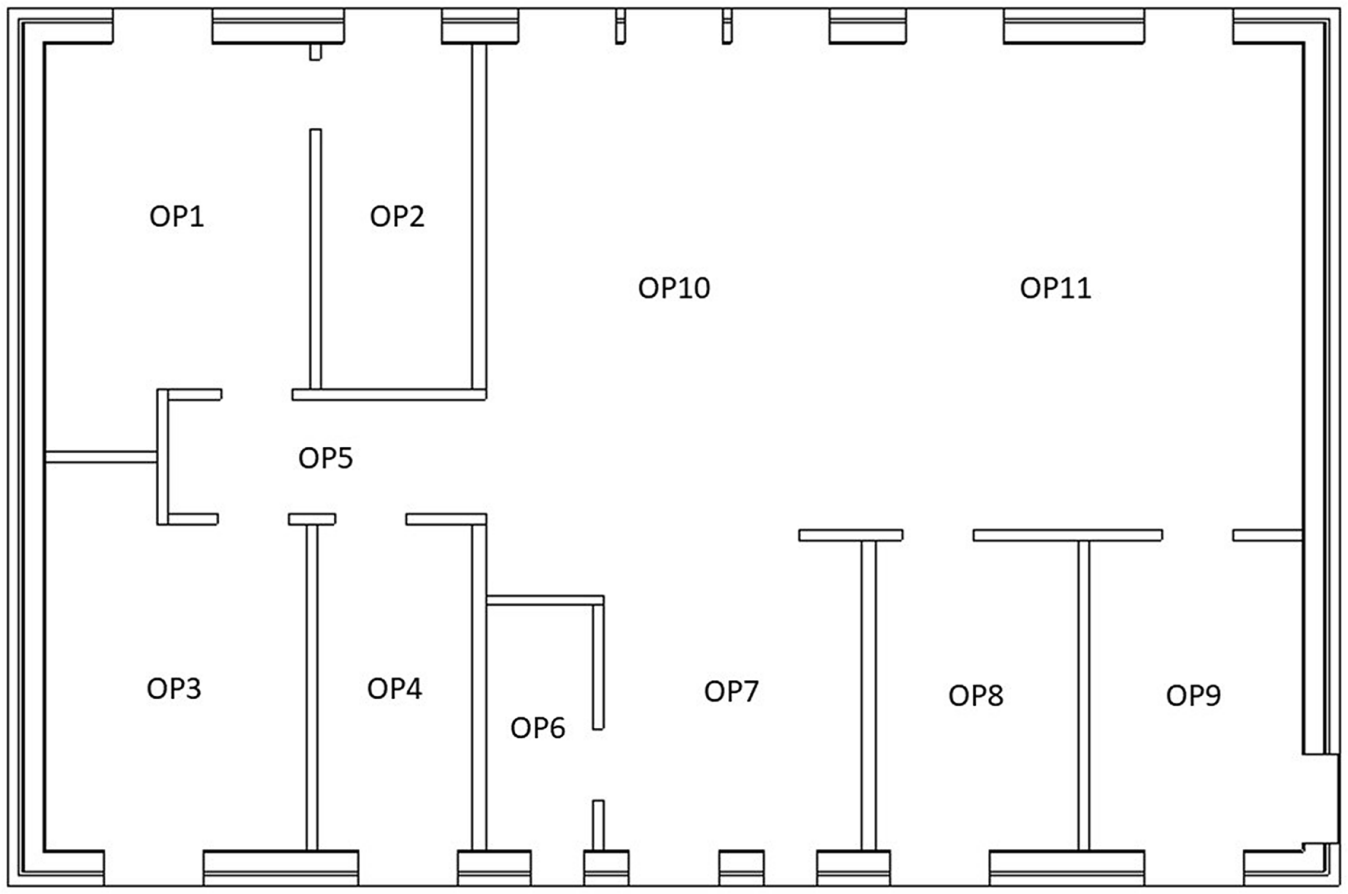
Observation points inside a typical Swedish house^6,7^.

The detector regions were defined as air-filled spheres with a diameter of 30 cm, positioned 1 m above ground level on properties P12–P15. These were located according to the observation points (OP) in our previous studies^6,7^ in the middle of the different rooms inside the house (Fig. 8), and an observation point was added in the middle of the garden of the houses P12–P15 (Fig. 7). The numbers and energies of the gamma ‘particles’ passing through these detector regions were determined using the Monte Carlo code. The gamma fluence was transformed into air kerma free-in-air using conversion coefficients^12^.

To determine the amount of waste with regard to relative and absolute dose reduction, the isodose concept, developed in a previous study^5^, was applied. The application in detail was performed with the following steps. First, the property with the highest absolute, and thus also relative, dose contribution per size of the area to one observation point was determined. The absolute and relative dose reduction that would result from decontamination were then assigned to the volume of generated waste caused by topsoil removal of this area. The volume of waste was calculated by multiplying the size of the area by 5 cm, based on the recommended thickness for topsoil removal^3,4^. In all cases analysed here, the property with the highest absolute/relative dose contribution turned out to be the same property at the considered observation point. In the second step, the property with the second highest absolute/relative dose contribution per size of the area to the same observation point was determined. Then, the sum of the first and second highest absolute/relative dose reductions if these areas were to be decontaminated was assigned to the total volume of generated waste caused by topsoil removal of both properties. This step was repeated until the sum of dose contributions from all 15 properties was reached in connection with the volume of generated waste for topsoil removal of all properties. The remaining dose contributions from the surfaces of the street and surroundings were not analysed in further detail as the focus of this study is on the effectiveness of topsoil removal in a limited suburban neighbourhood.

## Data Availability

The datasets generated during and analyzed during the current study are available from the corresponding author on reasonable request.
